# Editorial: New drugs, approaches, and strategies to combat antimicrobial resistance

**DOI:** 10.3389/fphar.2023.1295623

**Published:** 2023-11-06

**Authors:** Chandra Kant Singh, Kushneet Kaur Sodhi, Mohammad S. Mubarak

**Affiliations:** ^1^ Department of Zoology, University of Delhi, New Delhi, India; ^2^ Department of Zoology, Sri Guru Tegh Bahadur Khalsa College, University of Delhi, New Delhi, India; ^3^ Department of Chemistry, The University of Jordan, Amman, Jordan

**Keywords:** antimicrobial resistance, antibiotics, bacteria, repurposed medications, new drugs

Multidrug-resistant organisms (MDROs) are microorganisms, mainly bacteria, that have become resistant to one or more classes of antimicrobial agents and to certain antibiotics. Thus, antibiotics can no longer be employed to kill these microorganisms. MDROs, include but are not limited to, methicillin-resistant *Staphylococcus aureus* (MRSA), vancomycin-resistant Enterococci species (VRE), carbapenemase-producing *Enterobacteriaceae*, and Gram-negative bacteria that produce extended spectrum beta-lactamases (ESBLs). They also include *Escherichia coli* and *Klebsiella pneumoniae*, *Acinetobacter baumannii*, and organisms such as *Stenotrophomonas maltophilia* (Siegel et al., 2006). According to the World Health Organization (WHO), MDROs are a growing threat and pose a significant public health risk worldwide (Chan, 2017). Multidrug-resistant bacterial pathogens are an ultimate threat in today’s scenario, which requires an imperative necessity for novel policies to tackle bacterial infections. The multi-drug resistant *Pseudomonas aeruginosa* and *S. aureus* infect two million individuals as reported by U.S. Centres for Disease Control and Prevention [CDCP] (2013) (Chambers and Deleo, 2009).

In a therapeutic setting, antimicrobial resistance refers to a microbe’s capacity to prevent a medicine from acting against it. If nothing is done about it, by 2050 it will be the main cause of mortality. Bacteria have an adaptive mechanism that helps them develop and endure under challenging circumstances. Antibiotics are one such stressor; it has been found that numerous bacteria flourish in an environment tainted with antibiotics. The presence of resistance determinants in the bacterium is the primary cause of the organism’s capacity to survive antibiotic stress. Bacteria have both acquired and intrinsic properties to counter antibiotic stress. Since bacteria naturally synthesize antibiotics and antibiotic-resistant enzymes, it makes sense that the synthesis and resistance mechanisms would co-evolve. In soil, where the microbes that produce antibiotics coexist with other organisms, resistance to the antibiotics develops due to increased evolutionary pressure (Iskandar et al., 2022). The resistance determinants present in the bacteria-producing antibiotics that have orthologs in clinical isolates lend credence to the concept. Antimicrobial resistance has become a major menace owing to the irrational use of antibiotics in human medicine, agriculture, and veterinary. When bacteria are exposed to antibiotics in the environment, a selective pressure develops in the bacteria that causes the evolution of genes for antibiotic resistance.

This Research Topic on “*New drugs, approaches, and strategies to combat antimicrobial resistance*” discusses how the menace caused by antimicrobial resistance is complicated further as there is an increased resistance in bacteria against many topical antibiotics. We received multiple submissions on the Research Topic, of which four articles were included after rigorous peer review. These articles cover the latest developments in the field of antimicrobial resistance, ranging from the antibacterial activity of kernel extracts to the use of medicinal plants in the treatment of various ailments such as cancer. They include a research article describing the role of uracil in enhancing the gentamicin activity and helping in the killing of Gram-positive bacteria and an article describing the potential of whole genome sequencing of the bacteria *Alcaligenes* sp. Strain MMA is a powerful tool used to monitor the spread of antimicrobial resistance. The main reason for concern is that there has not been an antibiotic approved in a while to treat Gram-negative bacteria, and antibiotic testing is quite expensive. Bacteriophage treatments, bacteriocins, transition metal complexes, and peptide nucleic acids (PNAs), combined with CRISPR-Cas constructs can be used as effective antimicrobial agents to replace antibiotic therapy. Antibiotic resistance issues can also be resolved with the use of metagenomic research (Awasthi et al., 2022). According to research findings, repurposed medications have shown promise in treating a variety of disorders. The use of repurposed medications has recently swamped scientific publications, particularly during the COVID-19 era when a global medicine scarcity threatened. Drug repurposing is used to speed up the traditional method of drug discovery by evading the necessity for toxicity testing for medications that have been proven to be effective and safe in humans and approved by the FDA (Food and Drug Administration, United States) for other indications (Walsh et al., 2023).

In this Research Topic, a paper by Sodhi et al. showed that bacterial whole genome sequencing facilitates the study of antibiotic resistance, and high-throughput sequencing technologies can undoubtedly pave the path for more effective antibiotic resistance management. In addition, research by Fan et al. describes that uracil synergizes with gentamicin and regulates the tricarboxylic acid (TCA) cycle of the bacteria, thus aiding in killing Gram-positive bacteria. Moreover, the review article by Kong et al. describes the use of *Dendrophthoe falcata* (L.f.) Ettingsh. (DF) and *Dendrophthoe pentandra* (L.) Miq. (DP) for the treatment of a wide range of disorders, including cancer, ulcers, asthma, paralysis, and skin conditions. The authors reiterated the pharmacology, and phytochemistry along with their toxicity and traditional uses. Another review article by Bhaskaracharya et al. outlines the recent knowledge regarding the antibacterial activity of polyphenolic extract from *Phoenix dactylifera* (date kernel). The review highlights that date kernel extracts have a diverse antibacterial activity and show weak to moderate activity against Gram-negative bacteria and high to moderate activity against Gram-positive bacteria.

In summary, antimicrobial resistance (AMR), has grown to be the most feared threat, endangering world health. One of the biggest global public health issues now is the rise of antibiotic-resistant microorganisms (World Health Organisation, 2014). According to the World Health Organisation (WHO), the primary causes of AMR are antibiotic overuse and abuse. Recent research has demonstrated the brewing public health catastrophe with growth in resistance to carbapenems and polymixins, the last line of defense against antibiotics (Gandra et al., 2023). Within this context, 4.95 million people died worldwide from drug-resistant illnesses in 2019, of which 1.27 million fatalities were specifically attributed to AMR. To prevent another worldwide pandemic and address the issues surrounding antibiotic abuse and overuse, action is urgently required.
